# Benign pulmonary metastasizing leiomyoma of the uterus: A case report

**DOI:** 10.3892/ol.2015.2878

**Published:** 2015-01-15

**Authors:** HUI MA, JIE CAO

**Affiliations:** Respiratory Department, Tianjin Medical University General Hospital, Tianjin 300052, P.R. China

**Keywords:** lung, leiomyoma, benign lesion, histological origin

## Abstract

Pulmonary benign metastasizing leiomyoma (BML), is characterized by multiple pulmonary nodular lesions and is a rare disease. The present study reports the case of a 45-year-old asymptomatic woman who underwent an excision of uterine leiomyoma 11 years previously. Chest computed tomography (CT) revealed multiple bilateral pulmonary nodules five months prior to admission, during a regular check-up. Intravenous levofloxacin (0.5 g/day) was administered for one week, which demonstrated no effect. Positron emission tomography combined with CT (PET/CT) revealed no evident radioactivity concentration. Due to the suspicion of metastasizing leiomyoma, video-assisted thoracoscopic surgery, with a wedge resection of the right pulmonary lesion, was performed. Post-operative pathological examination revealed the lesion to be a pulmonary leiomyoma accompanied by local necrosis. Immunohistochemical staining revealed that the lesion was positive for the expression of smooth muscle actin, desmin, estrogen receptor, progesterone receptor and B-cell lymphoma-2. Cytokeratin and epithelial membrane antigen were not expressed in the tumor cells. Staining for Ki-67 revealed expression of Ki-67 in ~1% of the spindle cells. The overall morphological and immunohistochemical features, accompanied by the remote patient history of primary uterine leiomyoma, supported the diagnosis of pulmonary BML.

## Introduction

Pulmonary benign metastasizing leiomyoma (BML) is a rare disease that is commonly thought to develop years after the diagnosis and resection of uterine leiomyoma. The majority of BML patients reported in the literature possess a previous history of uterine leiomyoma resectioning, with the time period between uterine leiomyoma resectioning and nodule detection varying between three and 20 years (1). The majority of cases of BML reported in the literature presented as diffuse bilateral pulmonary nodules, occasionally accompanied by multiple pleural or peritoneal nodules (2). The main metastatic site of BML is the lung, of which the majority are bilateral nodules, 17% are unilateral nodules and 13% are solitary nodules (3), however, extrapulmonary lesions have been identified in the skin, greater omentum, inferior vena cava, right atrium of the heart, pelvis, muscle and brain (2,4). The present study reports an asymptomatic presentation of BML, in which histological examination, immunohistochemistry studies and clinical history correlation were performed to support the diagnosis. Written informed consent was obtained from the patient.

## Case report

**Figure 1:**
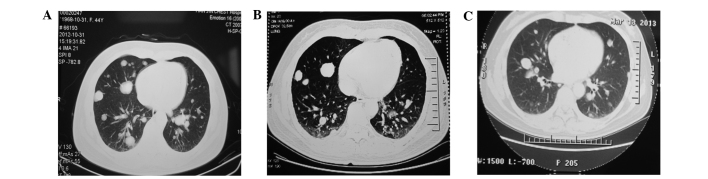
Chest computed tomography (CT) findings from different examinations. (A) CT revealing multiple variable-sized bilateral pulmonary nodules and a soft-tissue mass in the basal segment of the left-upper and bilateral lower lobes on Oct 31, 2012. (B) CT revealing that the multiple variable-sized bilateral pulmonary nodules were not reduced in size subsequent to therapy. The image was obtained on Nov 12, 2012. (C) CT revealing that the multiple variable-sized bilateral pulmonary nodules and lumps were increased in size on Mar 13, 2013.

The present study reports the case of a 45-year-old female who was referred to the Tianjn Medical University General Hospital (Tianjin, China) in March 2013 for the further examination of multiple bilateral pulmonary nodules that were incidentally identified on imaging during a regular check-up five months prior to the referral, on October 31, 2012. The patient was asymptomatic prior to hospitalization. Chest computed tomography (CT) was performed at Tianjin Chest Hospital (Tianjin, China), which revealed multiple variable-sized bilateral pulmonary nodules and a soft-tissue mass in the basal segment of the left upper and bilateral lower lobes (Fig. 1). The patient was subsequently administered intravenous levofloxacin (0.5 g/day) for one week. The results of the pulmonary function testing and the bronchoscopic examination were normal. Positron emission tomography combined with CT (PET/CT), performed at Tianjin Medical University Cancer Institute and Hospital (Tianjin, China), revealed an abnormal fluorodeoxyglucose uptake, with the suspicion of multiple variable-sized bilateral pulmonary nodules and lumps, and no evident radioactivity concentration was revealed. The patient was re-examined every two months using pulmonary CT due to the suspicion of metastasizing leiomyoma. No additional drugs were administered to the patient as no clinical symptoms were observed.

Five days prior to admission, an enhanced chest CT at the Tianjin Medical University General Hospital revealed multiple variable-sized bilateral pulmonary nodules and lumps. Certain nodules demonstrated significant enhancement, and other regions were attached to intrapulmonary vessels. Non-enlarged bilateral hilar lymph nodes, non-enlarged multiple small lymph nodes in the mediastinum and non-thickened bilateral pleura were observed. The quantity of nodules and lumps had increased compared with the PET/CT results obtained four months previously, which indicated a metastatic disease, without the exclusion of granulomatous angiitis (Fig. 1). An aspiration biopsy was performed for further diagnosis. The patient had experienced uterine leiomyoma 11 years prior to the current presentation, for which a laparoscopic myomectomy had been performed. The previous health of the patient was reasonable, without any smoking, drinking or familial history of cancer.

The results of the clinical examination were unremarkable, and the results from the routine laboratory tests were as follows. The results of the routine blood, urine and stool tests and liver and kidney function were normal, as were the four checking categories of lung cancer (carcino-embryonic antigen, neuron specific enolase, squamous cell carcinoma antigen and serum cytokeratin 19 fragment levels), and the levels of terminal restriction fragment, carbohydrate antigen (CA) 19-9, CA242, CA153 and human epididymis protein 4. The results of immune complex testing and testing for rheumatoid and anti-neutrophil cytoplasm antibodies were normal. The level of the antibodies against *Mycoplasma pneumonia*, *Chlamydia pneumonia* and *Legionella pneumophila* were normal. The results of the 1–3-β-D polyglucosan and purified protein derivative tests revealed negative results. Using a blood gas analyzer, the erythrocyte sedimentation rate in the patient was found to be 11 mm/h, while the blood pH was 7.46, the partial pressure of carbon dioxide was 37 mmHg, the partial pressure of oxygen was 84 mmHg and the oxygen saturation was 97%. The lavage liquid consisted of 77.5% macrophages, 19.5% lymphocytes, 3% neutrophil granulocyte and 0% eosinophilic granulocytes. No bacteria, fungi or yeast were identified in the alveolar lavage fluid, and acid-fast staining did not yield a positive result. The pelvic scan revealed a normal uterine shape, a non-uniformity of signals in the muscular layer and multiple cervical cysts.

**Figure 2:**
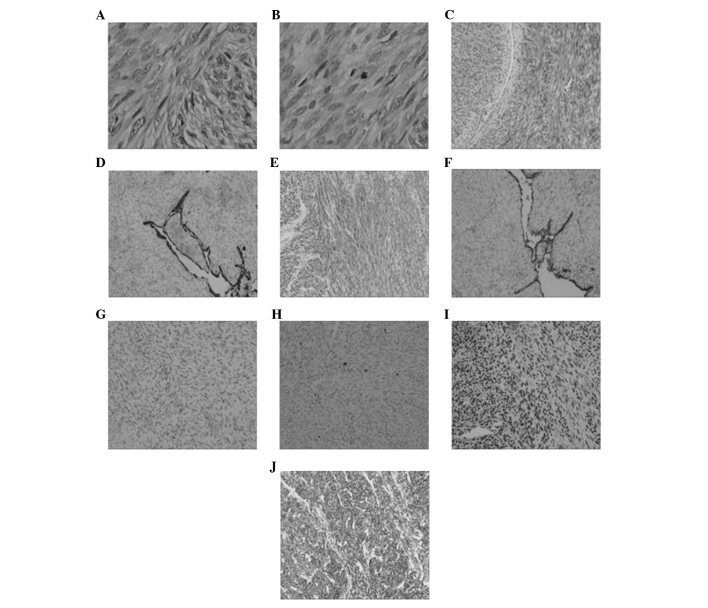
Pathological examination and immunohistochemical staining of benign metastasizing leiomyoma. (A and B) The tumor was composed of spindle-shaped muscle cells (maginfication, ×400). (C) The cells demonstrated positive immunohistochemical staining for B-cell lymphoma-2. (D) Cytokeratin (CK) staining revealed no CK expression in the cells. (E) The cells demonstrated positive staining for desmin, but (F) no epithelial membrane antigen was identified in the cells. (G) The cells were positive for estrogen receptor. (H) Ki-67 stained ~1% of the spindle cells, and the cells were positive for the expresison of (I) progesterone receptor and (J) smooth muscle actin expression.

A pulmonary wedge resection was performed on the right lung using video-assisted thoracoscopic surgery one week subsequent to admission. During the surgery, multiple nodules that were 1–2.5 cm at the maximum dimension were noted in each lobe of the right lung. Certain nodules extended to the two pleural surfaces. A hard and oval-shaped lump located in the middle and lower lobes was 3 cm in length and exhibited a similar appearance to the pulmonary surface, which connected with the right pulmonary lower lobe via the pleural surface. Intra-operative frozen section examination revealed that the lung wedge specimen was composed of three hoary nodules that were 3×2.8×1.5 cm, 1.2×1.2×0.7 cm, and 0.7×0.6×0.5 cm in size. However, BML in the right pulmonary lower lobe was accompanied by local necrosis. Immunohistochemical studies revealed that these cells were positive for the expression of smooth muscle actin, desmin, estrogen receptor, progesterone receptor and B-cell lymphoma-2, while there was no expression of cytokeratin or epithelial membrane antigen. Staining for Ki-67 revealed expression in ~1% of the spindle cells. These findings were consistent with benign metastasizing leiomyoma that developed from the known primary uterine leiomyoma (Fig. 2). The patient was discharged without any early post-operative complications. After five months of follow-up the general condition of the patient was satisfactory, without any radiological evidence of recurrent disease or distant metastases. Patient follow up is ongoing.

## Discussion

BML occurs mainly in premenopausal and perimenopausal females with a mean age of 47 years old (range, 30–74 years old). All BML patients reported in the literature possess a previous history of resectioning for uterine leiomyoma, with the time period between uterine leiomyoma resectioning and nodule detection varying between three and 20 years (1). The radiological presentation of BML is diffuse bilateral pulmonary nodules (2), occasionally accompanied by multiple pleural or peritoneal nodules. The main metastatic site of BML is the lung, of which the majority are bilateral nodules, 17% are unilateral nodules and 13% are solitary nodules (3), but extrapulmonary lesions have been documented in the skin, greater omentum, inferior vena cava, right atrium, pelvis, muscle and brain (2,4). The majority of patients with BML are asymptomatic and are diagnosed during physical examinations. The severity of the symptoms is strongly correlated with the size and quantity of nodules. Approximately one-third of patients present with cough, dyspnea and chest pain, and progressively develop respiratory insufficiency and failure, eventually succumbing to the disease. There are three clinical types of BML, according to the tumor position: a) BML in the pulmonary interstitium accompanied by chest tightness and dyspnea during tumor growth, which is common and asymptomatic; b) BML in the endobronchial lesions resulting in a non-specific, irritating cough and recurrent obstructive pulmonary emphysema at the early stage, which is not as common as the aforementioned type; and c) BML in the pulmonary vessels with repeated hemoptysis, which is rare. The patient in the present study possessed BML of the pulmonary interstitium.

The nature and etiology of BML remain controversial. Certain researchers hypothesize that BML is a type of multiple smooth muscle *in situ* proliferation that is induced by estrogen and progesterone, while others support that BML results from the monoclonal, hematogenous spread of a differentiated uterine leiomyoma (5). However, the majority of studies concur with BML being a metastatic leiomyoma that metastasizes between the uterus and the lung, due to all reported cases being in women with a previous history of uterine leiomyoma resectioning (1). The patient possessing multiple nodular lesions of various sizes in the bilateral lungs reported in the present study had undergone excision of uterine leiomyoma 10 years previously. Certain studies have reported cases with BML accompanied by multiple pleural or peritoneal nodules. In addition, the majority of recent findings are also consistent with the hypothesis of a monoclonal origin of the uterine and pulmonary tumors (6). All these studies indicate that pulmonary leiomyoma is metastatic. However, BML has been determined to be a benign lesion as these tumors consist of well-differentiated, benign-appearing smooth muscle cells with a regular karyotype that lacks pleomorphism or mitotic figures. However, additional study is required to determine the existence of primary pulmonary leiomyoma, as leiomyoma arising in men and children has been reported as non-metastatic, with residual alveoli in the lesions and no vascular tumor thrombus in the lung (7).

The majority of patients with BML possess a good prognosis, with the median survival time of 94 months (range, 6–101 months) subsequent to the excision of the intrapulmonary lesions (8). Schneider *et al* reported 10 cases with a median follow-up duration of 4.7 years (9). No local complications occurred, and no patients succumbed to BML. However, mortality is reported in certain cases. Therefore, BML is a borderline tumor with benign histological features, despite the biological behavior indicating malignancy (10).

In conclusion, although BML is a rare condition, it should be considered during the diagnosis of asymptomatic females of reproductive age with a history of uterine leiomyoma that present with solitary or multiple pulmonary nodules.

## Abbreviations

BML: benign metastasizing leiomyoma
CT: computed tomography
PET/CT: positron emission tomography
SMA: smooth muscle actin
ER: estrogen receptor
PR: progesterone receptorl
